# Assessment of digestates prepared from maize, legumes, and their mixed culture as soil amendments: Effects on plant biomass and soil properties

**DOI:** 10.3389/fpls.2022.1017191

**Published:** 2022-12-13

**Authors:** Tereza Hammerschmiedt, Antonín Kintl, Jiri Holatko, Adnan Mustafa, Tomas Vitez, Ondrej Malicek, Tivadar Baltazar, Jakub Elbl, Martin Brtnicky

**Affiliations:** ^1^ Department of Agrochemistry, Soil Science, Microbiology and Plant Nutrition, Faculty of AgriSciences, Mendel University in Brno, Brno, Czechia; ^2^ Agricultural Research, Ltd., Troubsko, Czechia; ^3^ Agrovyzkum Rapotin, Ltd., Rapotin, Czechia; ^4^ Institute of Chemistry and Technology of Environmental Protection, Faculty of Chemistry, Brno University of Technology, Brno, Czechia; ^5^ Institute for Environmental Studies, Faculty of Science, Charles University in Prague, Praha, Czechia; ^6^ Department of Agricultural, Food and Environmental Engineering, Faculty of AgriSciences, Mendel University in Brno, Brno, Czechia; ^7^ Department of Experimental Biology, Section of Microbiology, Faculty of Science, Masaryk University, Brno, Czechia; ^8^ Department of Agrosystems and Bioclimatology, Faculty of AgriSciences, Mendel University in Brno, Brno, Czechia

**Keywords:** waste management, agriculture, organic fertilizer, microbial activity, nutrient cycling

## Abstract

Digestate prepared from anaerobic digestion can be used as a fertilizer, as it contains ample amounts of plant nutrients, mainly nitrogen, phosphorous, and potassium. In this regard, digestates produced from mixed intercropped cereal and legume biomass have the potential to enrich soil and plants with nutrients more efficiently than monoculture-based digestates. The objective of this study was to determine the impact of different types of digestates applied at a rate of 40 t·ha^-1^ of fresh matter on soil properties and crop yield in a pot experiment with lettuce (*Lactuca sativa*) as a test crop. Anaerobic digestion of silages was prepared from the following monocultures and mixed cultures: broad bean, maize, maize and broad bean, maize and white sweet clover, and white sweet clover. Anaerobic digestion was performed in an automatic custom-made system and applied to the soil. Results revealed that fresh and dry aboveground biomass as well as the amount of nitrogen in plants significantly increased in all digestate-amended variants in comparison to control. The highest content of soil total nitrogen (+11% compared to the control) and urease (+3% compared to control) were observed for maize digestate amendment. Broad bean digestate mediated the highest oxidizable carbon (+48%), basal respiration (+46%), and N-acetyl-β-D-glucosamine-, L-alanine-, and L-lysine-induced respiration (+22%, +35%, +22%) compared to control. Moreover, maize and broad bean digestate resulted in the highest values of N-acetyl-β-D-glucosaminidase and β -glucosidase (+35% and +39%), and maize and white sweet clover digestate revealed the highest value of arylsulfatase (+32%). The observed differences in results suggest different effects of applied digestates. We thus concluded that legume-containing digestates possibly stimulate microbial activity (as found in increased respiration rates), and might lead to increased nitrogen losses if the more quickly mineralized nitrogen is not taken up by the plants.

## Introduction

Anaerobic digestion (AD) is a microbially-controlled process of biomethanization. Originally it was used for processing of biodegradable waste from agriculture, industry, or households (Angelidaki et al., 2003). Due to increasing threat of climate change and limited fossil energy sources, the bioenergy obtained from biogas has become essential in climate change mitigation, energy security, resourcing, and sustainable agriculture development (Shakoor et al., 2020). Research on the processing of liquid animal manure and plant raw material by AD has been promoted and expanded across the world (Aravani et al., 2022). Especially in Europe, biogas production from energy crops has developed considerably, and maize (*Zea mays* L.) used to be the most common and preferred source crop (Weiland, 2010) until recently. The advantages of maize biomass as substrate are high yield performance and established crop management technology (Gissén et al., 2014; Purdy et al., 2017; Theuerl et al., 2019). However, scientists have recently been looking for possible new substrates (Dębowski et al., 2022), mainly because of the negative environmental impacts and economic balance associated with maize cultivation (von Cossel et al., 2021; Herman et al., 2022).

Apart from the main product’s renewable energy (biogas), AD converts plant biomass to the second useful by-product—digestate. The resultant digestate has been characterized as having high amounts of plant-available nutrients and can act as a soil conditioner (Liedl et al., 2004; Arthurson, 2009; Brtnicky et al., 2022) or can be used as substrate for microalgae cultivation (Bauer et al., 2021; Kisielewska et al., 2021). Compared to the raw feedstock, the digestate obtains beneficial properties during AD, e.g., the increased availability of nitrogen mainly in NH_4_^+^ form (Gutser et al., 2005), potassium (Slepetiene et al., 2020) and phosphorus (Barłóg et al., 2020) for plants. The composition of digestate also depends on the raw materials being used as AD feedstock; for example, pig slurry contains more potassium (Zhan et al., 2020), whereas co-digested cattle slurry increased the content of phosphorus (Bachmann et al., 2011). Therefore, digestate (as an organo-mineral fertilizer) can provide comparably or almost as rich a nutrient supply as either mineral (Riva et al., 2016; Šimon et al., 2016) or organic fertilizers such as manure (Alburquerque et al., 2012; Bougnom et al., 2012).

So far, only a few studies have been focused on the quality of final digestate that are further governed by the type of feedstock (Risberg et al., 2017; Ehmann et al., 2018; Szymanska et al., 2018). However, substitutional crops are promising alternatives to biogas and digestate production from maize monocultures (Lebuhn et al., 2008; Oslaj et al., 2010; Sigurnjak et al., 2017; Gissén et al., 2014). Substitutional crops can be used either as co-substrates only (Meyer et al., 2015; Nurk et al., 2016; Fahlbusch et al., 2018; von Cossel et al., 2020) or blended biomass harvested from mixed cultures, such as maize and intercropped sunflower (Karpenstein-Machan, 2005; Nassab et al., 2011), sorghum (Schittenhelm, 2010; Samarappuli and Berti, 2018), or legume (Gatta et al., 2013; Kintl et al., 2019). Alternatively, maize could be fully replaced by another bioenergy crop of a common type (e.g. sugar beet, wheat, hemp) (Nges et al., 2012; González-García et al., 2013; Gissén et al., 2014). Moreover, the intercropping of energy crops with legumes represents a promising approach. The main advantage is the contribution of leguminous species to the nitrogen nutrition of non-leguminous species (Råberg et al., 2017). Legume biomass itself tends to have higher nitrogen content and lower C:N ratio than non-leguminous material (Peoples et al., 1995). A low C:N ratio changes the stability and consumption of available N nutrients, leading to slower nitrogen utilization, followed by higher alkalinity, *via* ammonia metabolism (Muhayodin et al., 2021). The higher content of ammonium from legumes in the intercrop biomass could inhibit the production of methane in biogas during AD (Wahid et al., 2018, Kintl et al., 2022). According to Hutnan et al. (2010), the process of anaerobic digestion is unstable at low N content in maize silage, and they recommend the addition of a substrate with higher nitrogen content for stabilization. Nevertheless, down-shifted AD performance and efficiency is compensated by higher residual nitrogen in the final digestate. The significantly negative correlation between cumulative nitrogen mineralization and the C:N ratio in digestate (Tambone and Adani, 2017) is indicative of improved digestate-derived nitrogen availability for plants in the amended soil (Barłóg et al., 2020), which is expected to further increase with increasing nitrogen content in the digestate (Andruschkewitsch et al., 2013). Another study indicated that reallocation of processed plant biomass (crop residues and cover crops) in the form of digestate can increase the crop dry matter yield, as well as nitrogen content in soil over a long term (Stinner et al., 2008). We assume that the use of digestate from cereal-legume mixed substrate as fertilizer could keep the subsequent crop biomass productivity consistently enhanced, as discussed by Råberg et al. (2017). Such an agro-system could become the future direction of sustainable agriculture and management of renewable sources (Kettl et al., 2010; Lamei Hervani, 2013).

The main objective of this study was thus to determine the impact of different types of digestates on soil properties and crop yield in a pot experiment with lettuce (*Lactuca sativa*). The feedstock used for digestate preparation was the biomass of either mixed crop (cereal-legume) cultures or monocultures (legumes, maize) varying in their chemical composition. We hypothesized that the soil amended with different digestates would contrast in: (I) final nutrient content (nitrogen, organic carbon) in soil, (II) the biomass yield of tested crop, and (III) soil microbial activities in relation to nutrient content in the digestates.

## Materials and methods

### Experimental field, plant biomass, and preparation of silages

**Figure 1:**
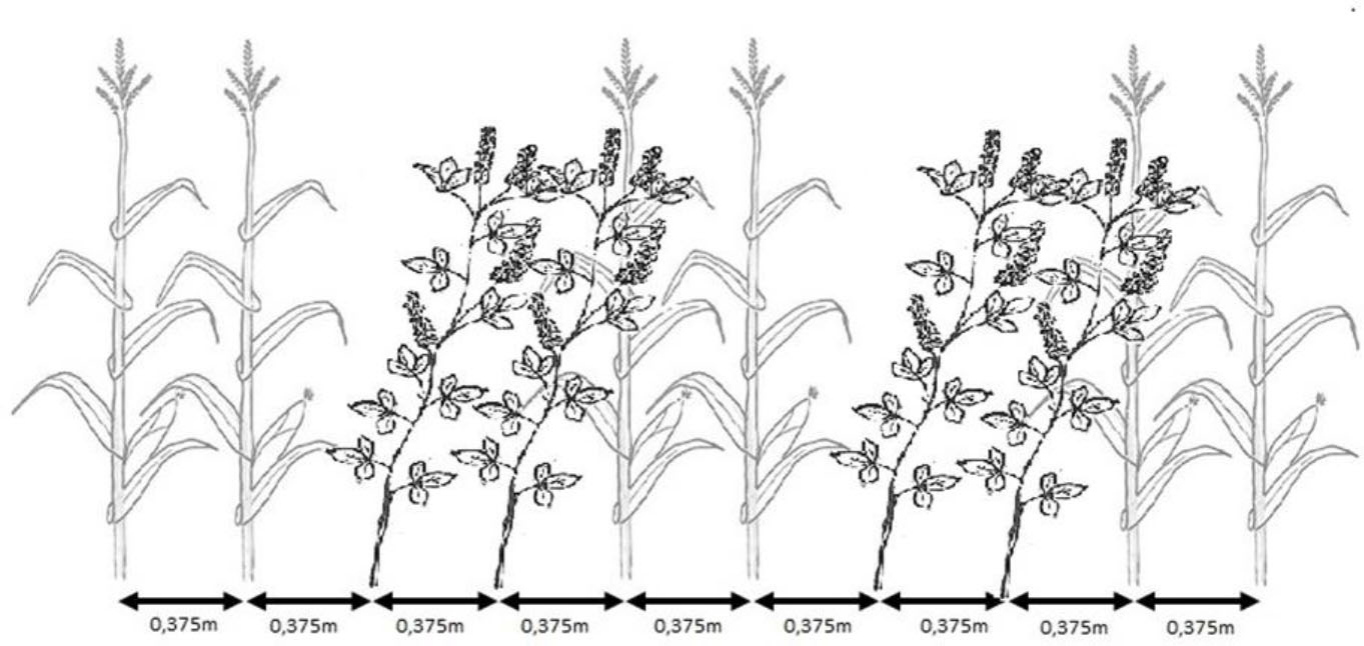
Scheme of sowing mixed culture of maize and selected legumes (Kintl et al., 2022).

The plants used for preparing digestates were grown at the Experimental Station for Fodder Crops in Vatin, Czech Republic (49°31’6”N 15°58’10”E). The station is located in a moderately warm area of the Bohemian-Moravian Highlands with a long-term average annual temperature of 7°C and a year-round long-term average rainfall of 658.6 mm; the values correspond to climate standards between 1981 and 2010 (Kintl et al., 2022). The soil of this site is characterized as sandy loam cambisol. The following variants were grown: a) monoculture of maize (*Zea mays* L.; BAYER, Ltd, Czech Republic); b) monoculture of broad bean (*Vicia faba* L. Amiga variety; Selgen a.s., Czech Republic); c) monoculture of white sweet clover (*Melilotus albus* MED., Meba variety - Research Institute for Fodder Crops, Ltd, Czech Republic); d) mixed culture of maize and broad bean; and e) mixed culture of maize and white sweet clover. The single crops as well as combination of maize and legume plants were sown by using the Kinze 3500 (Kinze Manufacturing, Williamsburg, IA, USA) “interplant system” seeding machine in a single operation (Kintl et al., 2019). Sowing was performed by alternating two rows of maize with two rows of legumes (**Figure 1**) to achieve a mixed culture system. The combination of maize and legume plants was sown at a rate of 150 thousand seeds per hectare, with maize and legume at the same rate of 75 thousand each. The monocultures were planted in half this sowing density at 75 thousand seeds per hectare with same row spacing (37.5 cm) as shown in **Figure 1**.

DASA fertilizer (300 kg·ha^-1^; DASA^®^ 26/13 Fertilizer CE, produced by Duslo corp., Slovakia; 18.5% w/w N-NH_4_, 7.5% w/w N-NO_3_, 13% w/w soluble S) was applied to all variants before sowing, in a dose that was sufficient to cover the nutritional requirements of maize and not limit the growth of legumes at the same time. The plant biomass was harvested at DM 35% (BBCH stage 77 – 83), determined on maize crop.

Sampling of plant biomass was performed manually at a height of 18 cm above the ground. Subsequently, a 15-20 mm cut was carried out using the Deutz-Fahr MH 6505 cutter (Deutz-Fahr, Lauingen, Germany). The cut was used to prepare model micro-silages in triplicate; see **Table 1**. The preparation of micro-silages was the same for all variants. Cut biomass (8 kg) was placed in a micro-silage container (Ø150 mm x 1000 mm) with inoculant (Silo Solve EF, CHr. Hansen, Denmark; *Lactococcus lactis*, *L. plantarum*, *Enterococcus faecium* - dose 6.25·10^5^ CFU per g of plant biomass), which equalled a dosage of 5 g·t^-1^ (of inoculant matter) + 3.5 L·t^-1^ H_2_O. The prepared inoculated cut biomass was compacted in micro-silage containers using a pneumatic press with a force of 6 N·m^-2^. Subsequently, the micro-silage container was sealed and placed in an incubation room without access to light at a constant temperature of 28°C ± 1°C for 90 days. At the end of the incubation period, the micro-silage containers were opened, silage was extracted and homogenized. Frozen samples of silages were transported to the laboratory to perform chemical analysis and fermentation tests.

**Table 1:** Prepared model silages.

Abbrev.	Variant	Weight content ofmaize fresh matter (%)	Weight content ofbroad bean fresh matter (%)	Weight content ofwhite sweet clover fresh matter (%)	Ratio
M	Maize	100	0	0	1
BB	Broad bean	0	100	0	1
WSC	White sweet clover	0	0	100	1
M+BB	M+BB	78.9	21.1	0	3.75:1
M+WSC	M+WSC	92.6	0	7.4	12.50:1

### Production of digestate

Anaerobic digestion of prepared silages was performed using fermentation batch tests in an automatic custom-made system consisting of 5 L glass fermenters placed in a heated water bath with adjustable temperature of 42°C ± 0.1°C. Each sample was fermented in triplicate. On the first day of the experiment, the fermenters were filled with 3 L of filtered (3 mm) inoculum obtained from agricultural biogas plant-processing maize silage and slurry (80/20, w/w%), operated at mesophilic conditions, with a hydraulic retention time of 80–90 days. The basic parameters of inoculum were as follows: total solids content 3.8%, volatile solids content 73%, pH 7.2, FOS 1452 mg∙L^-1^, TAC 4330 mg∙L^-1^, and FOS : TAC 0.34.

Initial organic loading rate was 5.5 g volatile solids of introduced substrate per L. The fermentation test was carried out until the daily biogas production in three consecutive days was < 1% of the total biogas production as stated in VDI 4630 (2016). This led to a retention time of 21 days. The digestate produced was subsequently analyzed to determine the following parameters. The dry matter was determined gravimetrically by desiccation at a temperature of 105°C ± 3.5°C to constant weight according to standard CSN EN 15934. TN (total nitrogen) and TC (total carbon) were measured using the Vario Macro Cube (Elementar Analysensysteme GmbH, Langenselbold, Germany). The content of total organic (TOC) and total inorganic (TIC) carbon was measured using Soli TOC^®^ Cube (Elementar Analysensysteme GmbH, Langenselbold, Germany). Basic nutrients (P, K) were extracted using the Mehlich III reagent and then analyzed using atomic emission spectroscopy (The Agilant55B AA, Agilent, CA, USA).

### Pot experiment

The following pot experiment investigated the effect of prepared digestates (**Table 2**) amendment on the soil properties and the growth of lettuce (*Lactuca sativa*). The experimental pots of volume 1 L were filled with 1 kg of experimental soil, which was prepared from silty clay loam (Haplic Luvisol) collected from a depth of 0-10 cm, sieved through a grid size of 2.0 mm to remove all roots and coarse particles, and mixed with fine quartz sand 0.1–1.0 mm (1:1 each, w/w). The content of nutrients in the topsoil used was as follows: total carbon (TC) 7.0 g·kg^−1^, total nitrogen (TN) 800 mg·kg^−1^, available phosphorus (P) 485 mg·kg^−1^, and available potassium (K) 0.115 mg·kg^−1^. The detailed properties of the soil used can be found elsewhere (Holatko et al., 2020; Brtnicky et al., 2021).

**Table 2:** Properties of digestates, dosage of nitrogen in 40 tons of the digestates per hectare.

Variant	Dry matter [%]	N [g∙kg^-1^ d.m.]	P [g∙kg^-1^ d.m.]	K [g∙kg^-1^ d.m.]	TOC [% d.m.]	TIC [% d.m.]	N dose [kg·ha^-1^]
M*	2.76 ± 0.06 ^ab^	152.17 ± 7.54 ^b^	22.01 ± 0.56 ^c^	6.52 ± 0.21 ^bc^	29.25 ± 0.01 ^d^	0.08 ± 0.00 ^c^	168.0 ± 11.8
BB*	2.49 ± 0.06 ^bc^	180.72 ± 8.36 ^ab^	23.29 ± 0.61 ^c^	8.83 ± 0.40 ^a^	28.80 ± 0.06 ^e^	0.10 ± 0.00 ^b^	180.0 ± 11.8
WSC	2.98 ± 0.14 ^a^	146.60 ± 9.10 ^b^	21.76 ± 0.65 ^c^	6.32 ± 0.26 ^c^	29.93 ± 0.05 ^b^	0.10 ± 0.00 ^b^	176.0 ± 11.8
M+BB*	2.94 ± 0.08 ^a^	159.86 ± 5.89 ^b^	31.63 ± 0.86 ^a^	7.82 ± 0.34 ^ab^	29.54 ± 0.05 ^c^	0.10 ± 0.01 ^b^	188.0 ± 9.8
M+WSC	2.23 ± 0.07 ^c^	205.70 ± 12.66 ^a^	28.18 ± 1.23 ^b^	8.50 ± 0.32 ^a^	30.21 ± 0.04 ^a^	0.13 ± 0.00 ^a^	184.0 ± 11.8

DM., dry matter; N, P, K, total nutrient (nitrogen, phosphorus, potassium) content; TOC, total organic carbon; TIC, total inorganic carbon. Different superscript letters indicate statistically significant differences between displayed values at the significance level p ≤ 0.05.

*, that the respective variants were presented in our previous paper Brtnicky et al. (2022).

The control variant was unamended, whereas digestates were applied to the other variants at a dose corresponding to 40 t∙ha^-1^; see **Table 2**. A total of 6 variants (control and 5 digestate variants) were made in 3 repetitions each.

Five sprouted seeds of lettuce (*Lactuca sativa*) were planted into each of the filled pots. Seedlings were reduced to one in each pot after 14 days. The pot experiment was conducted for 6 weeks under controlled conditions in a growth chamber Climacell Evo (BMT, Czech Republic) under the following conditions: 12-hour photoperiod, white illumination, light intensity 20,000 lx, day/night temperature 22/18°C, and relative atmospheric humidity 70%. Soil moisture was maintained at 65% water-holding capacity throughout the experiment. Pots were placed in the growth chamber using a randomized scheme and rotated weekly to ensure homogeneity of growing conditions.

### Plant and soil measurements and data collection

At the end of the experiment, the aboveground biomass (AGB) of individual seedlings was harvested, and the weight of fresh and subsequently dry (drying at 60°C) biomass was determined. Nitrogen content in harvested lettuce (Plant N) was measured using the Vario Macro Cube (Elementar Analysensysteme GmbH, Langenselbold, Germany). Nitrogen uptake by plants (N uptake) was calculated from dry weight of the plant and the nitrogen content in biomass. After harvest, a mixed soil sample from each pot was taken, homogenized, sieved (≤ 2mm), and air-dried for measuring pH, total nitrogen (TN), and oxidizable carbon (C_ox_); cooled to 4°C for respiration measurement; and freeze-dried for enzyme activity estimation. The TN was determined according to ISO_13878 1998 and C_ox_ according to ISO_14235 1998. Basal respiration (BR) and substrate-induced respirations (SIRs) were measured using a MicroResp (The James Hutton Institute, Scotland) device according to the method (Campbell et al., 2003). Substrate-induced respiration was measured after adding specific energy sources to the substrate: N-acetyl-β-D-glucosamine (NAG-SIR), L-alanine (Ala-SIR), L-lysine (Lys-SIR), and L-arginine (Arg-SIR). Enzymatic activities of β-glucosidase (GLU), N-acetyl-β-D-glucosaminidase (NAG), arylsulfatase (ARS), phosphatase (Phos), and urease (Ure) were measured according to ISO_20130 2018.

### Statistical analysis

Data processing and statistical analysis were carried out with the help of the statistical program R version 3.6.3. (R_CORE_TEAM 2020) together with the additional packages “ggplot2” (Wickham, 2016). Multivariate analysis of variance (MANOVA) and principal component analysis (PCA) with dependence of different treatments were used for modelling the relation between the soil properties and selected treatments with help of the additional packages “factoextra” (Kassambara and Mundt, 2017) and “FactoMineR” (Lê et al., 2008). One-way analysis of variance (ANOVA) and Tukey’s Honest Significant Difference (HSD) from package “agricolae” (Mendiburu 2020) at the significance level of 0.05 were used to detect the difference among the treatments. The factor level means calculation (with 95% confidence interval – CI) was carried out by using “treatment contrast”. Partial eta-squared (ηp2) from package “BaylorEdPsych” (Beaujean, 2012) was used to measure the effect size, and the Pearson correlation coefficient was applied to determine the linear dependence among soil properties.

## Results

### Soil chemical properties and aboveground biomass

**Figure 2:**
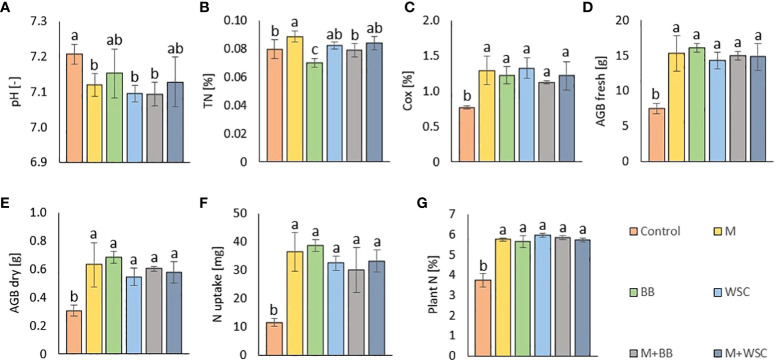
**(A)** Soil pH (n=6); **(B)** total nitrogen, TN (n=6); **(C)** total oxidizable carbon, C_ox_ (n=6); **(D)** fresh aboveground plant biomass, AGB fresh (n=3); **(E)** dry aboveground plant biomass, AGB dry (n=3); **(F)** nitrogen uptake by plant (n=3); **(G)** nitrogen content in plant biomass (n=9) of the control (no digestate) and all variants amended with digestates made from single crop and mixed cultures. Mean ± standard error of mean (error bars); different letters indicate statistically significant differences at the significance level p≤ 0.05.

**Figure 3:**
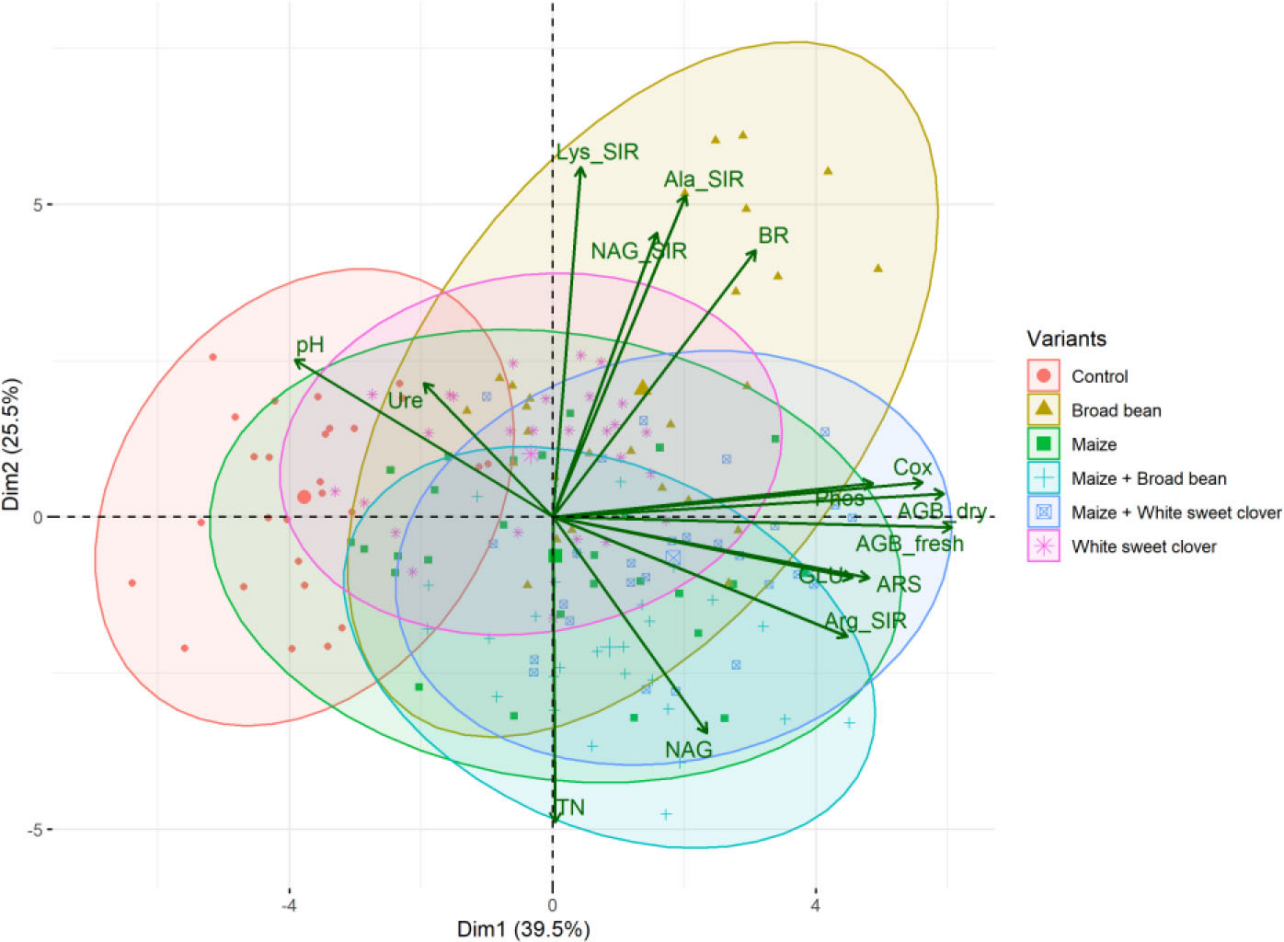
PCA biplot of relationships between soil and plant properties of all variants. Fresh (AGB_fresh) and dry (AGB_dry) aboveground plant biomass, soil pH, total nitrogen (TN), total oxidizable carbon (Cox), basal (BR), and substrate-induced respirations - N-acetyl-β-D-glucosamine (NAG_SIR), L-alanine (Ala_SIR), L-lysine (Lys_SIR), and L-arginine (Arg_SIR); enzyme activities - urease (Ure), arylsulfatase (ARS), N-acetyl-β-D-glucosaminidase (NAG), β-glucosidase (GLU), and phosphatase (Phos).

**Figure 4:**
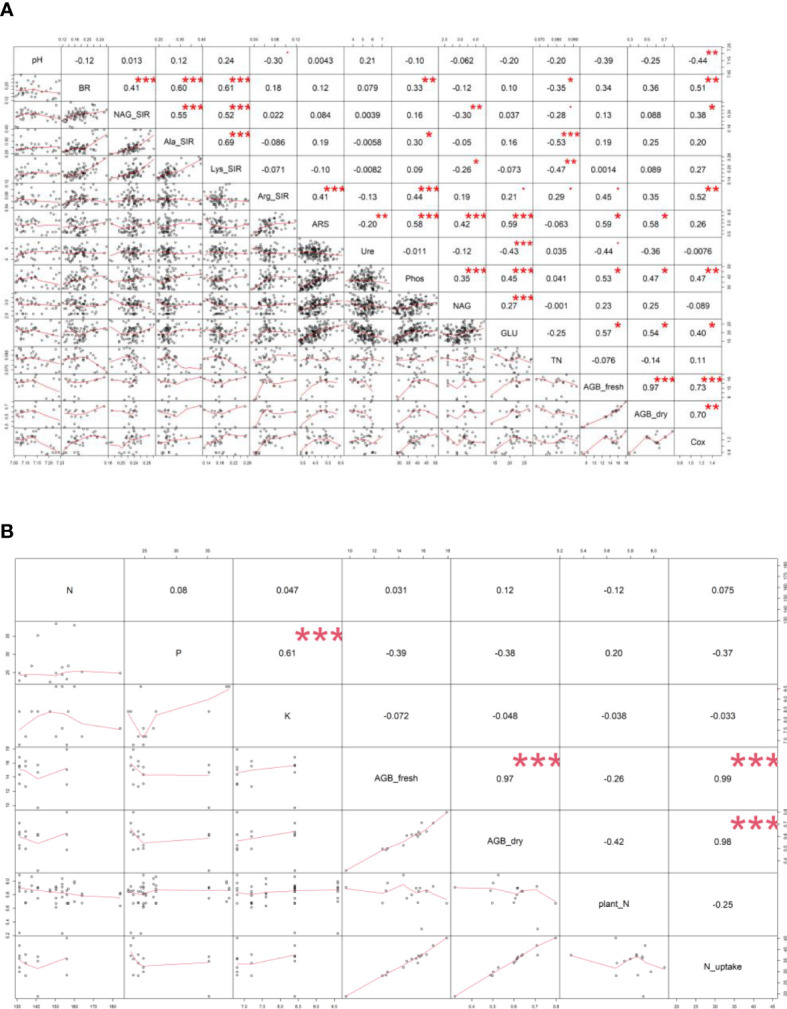
Pearson’s correlation analysis – values of correlation coefficient (r) between **(A)** soil and plant properties, **(B)** digestate (N, P, K content) and plant properties of all variants. Displayed correlation coefficients (r) were calculated on the statistical level of significance: * p ≤ 0.05, ** p ≤ 0.01, *** p ≤ 0.001. Fresh (AGB_fresh) and dry (AGB_dry) aboveground plant biomass, soil pH, total nitrogen (TN), total oxidizable carbon (Cox), basal (BR) and substrate-induced respirations - N-acetyl-β-D-glucosamine (NAG_SIR), L-alanine (Ala_SIR), L-lysine (Lys_SIR) and L-arginine (Arg_SIR), enzyme activities - urease (Ure), arylsulfatase (ARS), N-acetyl-β-D-glucosaminidase (NAG), β-glucosidase (GLU), phosphatase (Phos).

All the digestate-amended soils showed insignificantly different pH values compared to each other; the lowest average value of M+BB was 7.09; see **Figure 2A**. Also M, WSC, and M+BB digestates reduced soil pH significantly in comparison to the control. An antagonism was apparent between pH and Arg-SIR as well as NAG; see **Figure 3**. Further, pH was synergistic with Ure activity. The pH demonstrated significant (p ≤ 0.01) but low negative correlation with C_ox_ (r = - 0.44); see **Figure 4A**.

The highest soil TN (0.089%, in average) was detected in the M variant. On the other hand, BB variant showed the lowest TN value (0.07%). M, M+WSC, and WSC showed insignificantly different TN values (**Figure 2B**); all were significantly more TN abundant compared to the BB variant. TN correlated negatively and significantly (p ≤ 0.001) with Ala-SIR (r = - 0.53, **Figure 4A**) and showed an antagonism with other amino acid-induced respirations (Ala-SIR, Lys-SIR, NAG-SIR); see **Figure 3**.

The highest average C_ox_ was calculated in the WSC variant, but no statistical differences in values were found in any digestate-amended variants, which were significantly increased compared to the control; see **Figure 2C**. C_ox_ correlated positively and significantly with fresh AGB (p ≤ 0.001, r = 0.73) and with dry AGB (p ≤ 0.01, r = 0.70); see **Figure 4A**. Moreover, a significant (p ≤ 0.01) positive correlation was found with BR (r = 0.51) and Arg-SIR (r = 0.52). C_ox_ showed synergism with all mentioned traits and Phos, and an antagonism with pH and Ure.

Both fresh and dry AGB were significantly increased in all digestate-amended variants compared to the control. However, the differences between the amended variants were insignificant; see **Figures 2D, E**. The highest average fresh and dry AGB were achieved by fertilization with BB digestate, despite having the lowest value of TN and only the third highest C_ox_ content in the respective soil. Concurrently with lowest TN, nitrogen uptake by plant (N uptake) was on average (insignificantly) the highest in the BB variant (**Figure 2F**). Nevertheless, neither N uptake nor nitrogen content in dry AGB (Plant N) showed any significant differences between the digestate-amended variants; only the unamended control significantly had the lowest values of both properties; see **Figures 2F, G**. Fresh and dry AGB correlated significantly (p ≤ 0.001) positively and highly (r were 0.99 and 0.98) with N uptake; see **Figure 4B**. Both fresh and dry AGB correlated significantly (p ≤ 0.05) positively with enzyme activities, namely ARS (r was 0.59 and 0.58, respectively), Phos (r was 0.53 and 0.47, respectively), and GLU (r was 0.57 and 0.54, respectively); see **Figure 3**. Both fresh and dry AGB showed a strong synergism with these mentioned traits; see **Figure 3**.

### Soil respiration and enzymatic activities

**Figure 5:**
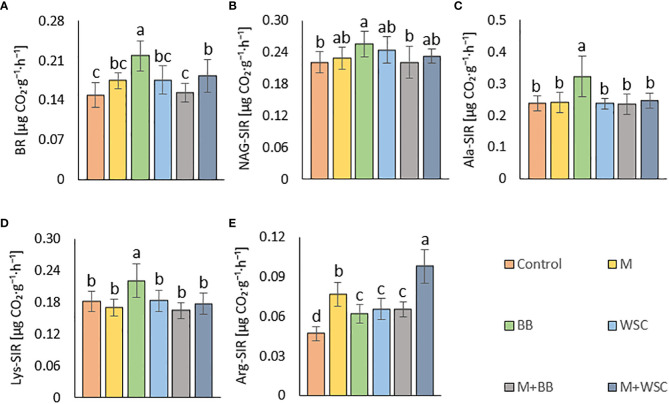
**(A)** Soil basal, BR and substrate-induced respirations - **(B)** N-acetyl- β -D-glucosamine, NAG-SIR; **(C)** L-alanine, Ala-SIR; **(D)** L-lysine, Lys-SIR; and **(E)** L-arginine, Arg-SIR; of the control (no digestate) and all variants amended with digestates made from single crop and mixed cultures, n=12. Mean ± standard error of mean (error bars); different letters indicate statistically significant differences at the significance level p ≤ 0.05.

Soil BR was significantly increased (compared to the control) only in two variants: BB (the highest average value, 219 µg CO_2_·g^-1^·h^-1^) and M+WSC; see **Figure 5A**. On the other hand, the soil fertilized with M+BB digestate and control exerted the significantly lowest BR. BR showed significant (p ≤ 0.001) positive correlation with NAG-SIR, Ala-SIR, and Lys-SIR (r was 0.41, 0.60, and 0.61, respectively); see **Figure 4A**. We revealed apparent synergism for four soil-respiration traits (BR, NAG-SIR, Ala-SIR, Lys-SIR); see **Figure 3**. NAG-SIR correlated significantly (p ≤ 0.001) and positively with Ala-SIR and Lys-SIR (r was 0.55 and 0.52, respectively); see **Figure 4A**.

BB was only variant which exerted significantly increased NAG-, Ala-, and Lys-SIR in comparison to the control; see **Figures 5B–D**. We detected comparable values of these SIR for all other variants. Mutual correlation between NAG- and Ala-SIR, NAG- and Lys-SIR, and Ala- and Lys-SIR was significant (p ≤ 0.001) and positive (r was 0.55, 0.52, and 0.69, respectively); see **Figure 4A**. Contrary to the other respiration traits, Arg-SIR was increased in all digestate-amended variants compared to the control; see **Figure 5E**. The amendment of M+WSC digestate showed the highest Arg-SIR. Arg-SIR correlated significantly (p ≤ 0.001) and positively with ARS (r = 0.41) and Phos (r = 0.44); see **Figure 4A**.

**Figure 6:**
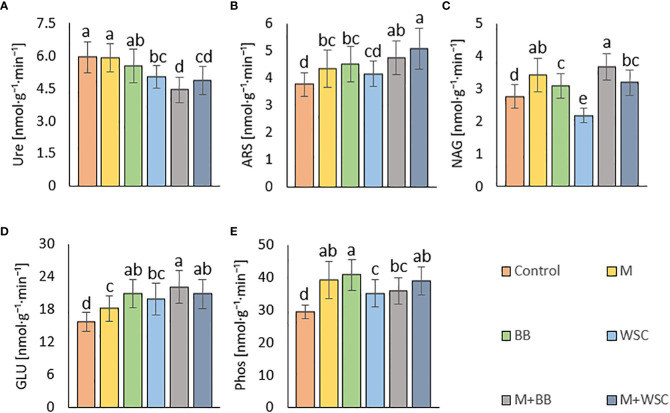
Soil enzyme activities - **(A)** urease, Ure; **(B)** arylsulfatase, ARS; **(C)** N-acetyl-β-D-glucosaminidase, NAG; **(D)** β-glucosidase, GLU; **(E)** phosphatase, Phos; of the control (no digestate) and all variants amended with digestates made from single crop and mixed cultures, n=27. Mean ± standard error of mean (error bars); different letters indicate statistically significant differences at the significance level p ≤ 0.05.

We detected the unaltered Ure activity (compared to the control) only in two variants amended with digestate: BB and M; see **Figure 6A**. The M variant showed significantly increased Ure activity in comparison to WSC, M+WSC, and M+BB (the lowest Ure value). A significant (p ≤ 0.001) negative correlation was found between Ure and GLU activity (r = - 0.43); see **Figure 4A**. PCA biplot showed antagonism with GLU, ARS, NAG activity, and Arg-SIR; see **Figure 3**.

Soil amended with M+WSC digestate exerted the significantly highest ARS activity, whereas amendment of WSC digestate resulted in comparably low ARS as detected in the control; see **Figure 6B**. ARS correlated highly significantly (p ≤ 0.001) and positively with GLU, Phos, and NAG activity (r was 0.59, 0.58, and 0.42, respectively); less significant (p ≤ 0.05) was the correlation of ARS and fresh AGB and dry AGB (r was 0.59 and 0.58, respectively); see **Figure 4A**. PCA biplot showed antagonism with GLU, ARS, NAG activity, and Arg-SIR; see **Figure 3**. The WSC variant revealed both the lowest ARS and lowest NAG from all digestate-amended variants; its NAG was significantly lower compared to the control; see **Figure 6C**. All other variants showed significantly increased NAG compared to the control, and M+BB variant had the significantly highest NAG.

The GLU and Phos activities of the control were significantly lowest compared to all other variants. GLU values of M and WSC variants were significantly lower in comparison to the highest M+BB variant; see **Figure 6D**. The highest Phos was revealed in the BB variant, which was significantly increased compared to the M+BB and WSC variants; see **Figure 6E**. GLU correlated significantly positively (p ≤ 0.001) with Phos (r = 0.45) and less significantly (p ≤ 0.05) with fresh AGB, dry AGB, and C_ox_ (r was 0.57, 0.54, and 0.40, respectively). Phos correlated significantly (p ≤ 0.05) with fresh AGB and dry AGB (r was 0.53 and 0.47, respectively) and (p ≤ 0.01) with C_ox_ (r = 0.47); see **Figure 4A**.

## Discussion

### Soil chemical properties and aboveground biomass

We discovered that adjustment of all digestates prepared from M, WSC, and M+BB decreased the average values of soil pH compared to the control’s unamended soil; see **Figure 2**. The respective digestates (M, WSC, and M+BB) exerted lower nitrogen content (on average 152.17, 146.60, and 159.86 g·kg^-1^ d.m., respectively) and potassium content compared to other two digestate types (**Table 2**). These digestate properties might have contributed to the observed differences in soil pH values.

The highest TN (0.09%) content in soil was detected under treatment with M-derived digestate, which was the only significantly increased value in comparison to the control. This finding did not correspond to the nitrogen doses applied to the soil with digestates. The M variant received a lower nitrogen dose compared to the nitrogen in BB and M+BB variants; see **Table 2**; however, these variants showed decreased TN in soil compared to the M digestate-amended soil. Because the nitrogen input did not correlate with N uptake by plants (**Figure 4B**), a possible explanation for this could be the lowered losses of nitrogen: lower pH putatively mediated reduction of ammonium to non-volatile cation form, and its volatilization was further mitigated due to enhanced nitrogen transformation and mineralization activities. A study done with slurry digestate showed that NH_3_ emissions were lower when it was acidified (Covali et al., 2021). Decreased TN content in the M+BB variant could have been coupled with decreased nitrogen mineralization, determined by urease (Ure) activity (compared to other legume-based digestate variants); see **Figure 6A**. Ure was comparable to the control only in M variant, in which the Ure value was significantly increased compared to WSC, M+BB, and M+WSC. A ratio of soil C:N (C_ox_ : TN) was also slightly different from the most favorable value, 20:1, which presented an assimilation:mineralization equilibrium value as 14.6:1 in M variant, which was lower in comparison to 17.6:1 in BB and 16.2:1 in WSC variants, indicating an M digestate-mediated higher nitrogen mineralization, as discussed by Brust (2019). We ascertained from these findings a higher nitrification rate in soil treated with M digestate, which was evidenced by high Ure value and significantly increased Arg-SIR compared to control; see **Figure 5E**. These assumptions were further corroborated by negative correlation between TN content and Ala-SIR (r = - 0.53, **Figure 4A**) and antagonism with Ala-SIR, Lys-SIR, and NAG-SIR, as displayed in PCA biplot; see **Figure 3**.

N-acetyl-β-D-glucosaminidase (NAG) was significantly increased in soil treated with M+BB variant compared to all other digestate variants; see **Figure 6C**. High NAG together with the (average) highest β-glucosidase (GLU) activity indicated enhanced turnover of soil fungal necromass and coupled nitrogen transformation, which could be also ascertained from the second lowest C_ox_ : TN ratio (14.3:1) of M+BB variant (Hu et al., 2015). With the enhanced turnover of putatively more abundant fungal biomass in the soil amended with the most phosphorus-rich digestate M+BB (31.63 ± 1.21 g·kg^-1^, **Table 2**), it was assumed that increment in fungal biomass was coupled with the respective digestate-derived improvement of phosphorus content in soil, as referred to by Du et al. (2019). On the other hand, a similar contributing effect to fungal biomass *via* higher access of phosphorus in soil could also be achieved by its mineral source, due to the fungal-derived improvement of phosphorus solubilization (Ceci et al., 2018).

All digestate-amended variants exerted significantly increased soil C_ox_ in comparison to the control variant. As C_ox_ represents a sub-pool of labile SOC, we expected that C_ox_ could correspond to a dose of TOC applied in the digestate to the respective variants. However, disproportions between C_ox_ and the applied dose of TOC were detected; see **Table 2** and **Figure 2C**. Although WSC showed the highest soil C_ox_ and the second highest TOC content in digestate, M+WSC reached the second lowest soil C_ox_ and concurrently the highest content of TOC in the respective digestate. The significant differences might have been caused by varied assimilation rate of carbon and nitrogen among the tested variants and could also be attributed to the digestibility of the organic carbon in the respective digestates, with higher lability of organic carbon in the fertilizer obtained from mono-substrate BB and WSC, M digestion. This assumption was based on the observed significantly longer lag in biogas production of the mixed culture feedstock compared to the monoculture corn feedstock (Kintl et al., 2022). We assumed vastly pre-digested, partially consumed (and therefore decreased to lower TOC values), and more easily available recalcitrant compounds in TOC of monoculture digestate, whereas the mixed culture digestate likely preserved a higher portion of moderately recalcitrant carbon. This might cause C_ox_ values to affect the respiratory and enzymatic activities in soil differently and incoherently with the carbon inputs derived by the various digestate variants. The efficiency of utilizing the digestate-derived external organic matter in the form of oxidizable carbon could be expressed as a ratio between TOC values of all digestate variants and C_ox_ values of respective amended variants used to treat soil. These ratios, calculated from **Table 2** and the values in **Figure 2C**, were also the lowest for WSC (22.5:1), M (22.6:1), and BB (23.4:1), compared to values of M+WSC (24.7:1) and M+BB (26.2:1). The microbial community amended with mixed culture digestate putatively oxidized such partially digested recalcitrant carbon more slowly and likely led to the significantly lowered respiration (BR, NAG-SIR, Ala-SIR, Lys-SIR) values in M+BB compared to BB variant; see **Figure 5**. The observed higher soil catabolic activity (BR, SIRs, GLU, NAG) in the respective monoculture digestate variants, namely BB, corroborates this presumption of higher organic matter decomposition in the monoculture digestate-amended variants. A contradictory significant positive correlation between C_ox_ and both fresh and dry AGB (r was 0.73 and 0.70, respectively) could also be explained by this hypothesis and the mutual synergy of these properties on the PCA biplot; **Figures 3**, **4**. An active pool of TOC could be also affected by the land use systems (Sahoo et al., 2019), but in this experiment the carbon sources in soil were much more affected by the addition of external organic matter of variable quality than by the changes in complexity of intrinsic SOM. Because C_ox_ correlated significantly and positively with BR (r = 0.51, p ≤ 0.01), we presumed that higher digestibility of digestate amendment enhanced organic matter aerobic decomposition. Moreover, TN content was coupled with increased respiration, evidenced by the significant negative correlation of TN and Ala-SIR and Lys-SIR (r were - 0.53 and – 0.47, p ≤ 0.001 and ≤ 0.01, respectively), and antagonism on PCA biplot; **Figures 3**, **4**. Therefore, a strong dependence between a decrease in nitrogen content in soil and the co-decomposition of nitrogen sources together with carbon sources and their mineralization was ascertained from these relations. Nevertheless, all digestate-amended variants significantly increased fresh and dry AGB in comparison to the control, and their effects remained similar to each other (**Figures 2D, E**). The highest average fresh and dry AGB value was achieved in BB digestate-treated variant despite the lowest value of TN and the third highest C_ox_ content in soil. It was reported that digestates have a higher potential over a short- to mid-term use period to positively affect plant biomass yield in comparison to synthetic nitrogen fertilizer under favorable climatic conditions (Doyeni et al., 2021). However, we discovered that plant nitrogen content (%) in plant dry biomass did not correspond to the amount of nitrogen amended with the respective digestate doses to the experimental variants. Nevertheless, we found a very high significant correlation of N uptake and fresh and dry AGB (**Figure 4B**), despite the differences between variants not being significant. Thus, soil nitrogen losses and N uptake at the end of the experiment are balanced through nitrogen access (provided by amended digestate) and acquisition by the plant. However, we can ascribe the rate of nitrogen efflux from soil to higher incorporation of nitrogen into plant biomass, as revealed in the BB variant, which showed the lowest soil TN and the highest average N uptake by plants.

### Soil respiration and enzymatic activities

Tested digestates consisting of M, M+BB, and WSC did not enhance the soil BR compared to the control; see **Figure 5**. Nevertheless, BR values of M,WSC, and M+WSC were comparable. We ascribed this finding to observation that the remaining organic fraction after AD was more recalcitrant than the feedstocks (Möller, 2015), thus the readily available carbon for aerobic catabolism was limited. The highest BR in BB-treated soil corresponded to the significantly lower content of stable (left intact after AD decomposition) carbonaceous compounds (hemicellulose, neutral detergent fiber) in legume-based digestate than in *Poales*-based digestate (Slepetiene et al., 2016). This was putatively caused by the reported higher (by +62%) inhibition of plant cell wall digestion by lignin in grasses than in legumes (Buxton and Russell, 1988). The significantly highest NAG-SIR in the BB variant was thought to be caused by higher degradable organic matter (fungal necromass) and other labile SOM-associated organic nitrogen supplied *via* respective legume-digestate amendment. Nitrogen scarcity anticipated the level of Ure activity as well, which was significantly increased in the variant BB (with the lowest TN) compared to all other legume-based variants (except WSC); see **Figures 2B**, **6A**. The soil treated with variants M+BB, M+WSC, and WSC with TN was comparable to the control value, and the C_ox_ : TN ratio was significantly lower (from 14.5:1 to 16.2:1) compared to the BB (17.6:1) soil; significantly decreased Ure compared to the control and M was also found. Retarded organic matter deamination in M+BB variant was again putatively caused by higher recalcitrance of external organic matter of digestate and indicated by low C_ox_:N ratio. Positive correlation between Ure activity and C:N ratio value was already reported (Gao et al., 2019; Pan et al., 2021).

It is generally known that fodder legumes are relatively less abundant in sulfur amino acids (Witten et al., 2015) compared to cereals such as maize. Thus, it was expected that the digestate made of single cropped BB and WSC might be deficient in sulfur. This assumption explained lower ARS (the enzyme involved in mineralization of organo-sulphates) in the WSC variant; see **Figure 6B**. The low ARS value in the M digestate-treated variant was less expected and comparable to the effect of BB digestate.

NAG is an enzyme involved in decomposition of fungal cell wall polysaccharide (chitin). Mycorrhizal fungi in the tested soil might have significantly affected the final fungal biomass and its turnover. Increment in fungal biomass was presumably coupled with the respective digestate-derived improvement of phosphorus content in soil, as discussed Du et al. (2019). The study by Yu et al. (2020) evidenced a negative correlation between N:P ratio and arbuscular mycorrhizal abundance and vesicle formation after root infection. This means that phosphorus supplementation by M+BB digestate with significantly lower N:P ratio (5.1:1) compared to other digestates (with N:P ratios demonstrably higher - from 6.7:1 to 7.8:1) may cause an increment in NAG values; see **Figure 6C**. On the other hand, WSC digestate dose exerted a higher N:P ratio of 6.7:1, which might partially explain the revealed lowest NAG although it was in contrast with the result of NAG-SIR determination. Our findings that NAG and ARS activity was related to the availability of nutrients in soil are further supported by the positive correlation of both enzyme activities with fresh and dry AGB.

The GLU enzyme catalyzes the hydrolysis of β-1→4-bonds in the cellulose and oligosaccharide molecules of soil organic matter (SOM) or digestate, especially its organic carbon fraction. Therefore, the significantly highest level of GLU in the M+BB variant corresponded to the highest dose of TOC in the respective applied digestate (372 kg·ha^-1^). Moreover, we assumed that digestates made of mixed cultures and monocultures of legumes contained more decomposition products of cellulose (hemicellulose) compounds due to the already mentioned digestion inhibition of cell wall compounds by lignin in grasses (Buxton and Russell, 1988) and thus induced higher GLU activity in comparison to the M digestate-amended variant.

We revealed the highest Phos activity in the BB variant, which was supplied with the lowest amount of available phosphorus (23 kg∙ha^-1^) within the amended digestate dose in comparison to all other digestate variants; see **Table 2**. We assume that the increased demand for phosphorus of lettuce seedlings exerting the highest fresh and dry AGB in the BB variant induced the enhanced phosphate-solubilizing activity in soil, catalyzed by the Phos enzyme. We discovered that the enhanced Phos activity was related to the increase in GLU values, and this was in the line with coupled carbon- and phosphorus-cycling enzyme activities (Loeppmann et al., 2020). On the other hand, the significantly lowest Phos in WSC was indirectly related to the highest TOC:P ratio (13.8:1) in the respective amended digestate calculated from **Table 2**. This finding agreed with a negative correlation of C:P ratio to phosphate solubilization efficiency as reported by Zhan et al. (2021).

## Conclusions

This study verified an apparently variable effect of digestates made from different feedstocks on soil properties. Fresh and dry aboveground biomass was significantly increased in the digestate-amended variants in comparison to the control but comparable after application of all five digestate types. The demonstrably highest content of total nitrogen and urease, and very high content of oxidizable carbon were observed for maize digestate amendment. However, the highest induction of respiration and enzyme activities occurred with the addition of digestates made of either legume monoculture or mixed cultures. We ascribe these observed differences to the effect of presumably increased soil degradability of legume-derived digestate organic matter and joint increased availability of nutrients after application, which might also lead to increased risks of nitrogen loss. Therefore, further investigation in this area could focus on the different ratios between maize and legume biomass in feedstock to achieve a better balance between the availability and recalcitrance of the digestate organic matter.

## Data availability statement

The original contributions presented in the study are included in the article/supplementary material. Further inquiries can be directed to the corresponding authors.

## Author contributions

Conceptualization, MB and TH; methodology, TH, AK, and AM; software, TB; validation, TB, JE, OM, and JH; formal analysis, JE and TH; investigation, AM; resources, JH and OM; data curation, TH, AM, and AK; writing - original draft preparation, TH, JH, AM, and MB; writing - review and editing, AK, AM, TV, and MB; visualization, TB and AM; supervision, MB, and TH; project administration, MB and AK; funding acquisition, JH, AK, and MB. All authors have read and agreed to the published version of the manuscript.
